# An Update on Unloading Knee Braces in the Treatment of Unicompartmental Knee Osteoarthritis from the Last 10 Years: A Literature Review

**DOI:** 10.1055/s-0038-1661382

**Published:** 2018-07-02

**Authors:** Dylan A. Mistry, Amit Chandratreya, Paul Y. F. Lee

**Affiliations:** 1 South Wales Orthopaedic Research Network, Cardiff University, Welshbone, Cardiff, Edwalton, Nottingham, United Kingdom; 2 South Wales Orthopaedic Research Network, Abertawe Bro Morgannwg University Health Board, Wales Hospital, Welshbone, Bridgend, United Kingdom; 3 Department of Trauma and Orthopaedic, LEO Institute, Grantham and District Hospital, United Lincolnshire Hospitals NHS Trust, Grantham, United Kingdom; 4 School of Sport and Exercise Science, University of Lincoln, Lincoln, Lincolnshire, United Kingdom

**Keywords:** knee osteoarthritis, braces, walking, knee arthroplasty, varus, valgus

## Abstract

**Background** The incidence of osteoarthritis is increasing and it is one of the most common causes of chronic conditions. Total knee replacement is the mainstay of treatment for end-stage knee osteoarthritis; however, with long waiting lists and high levels of dissatisfaction, a treatment like knee braces could potentially delay surgery. Unicompartmental knee osteoarthritis is associated with misalignment of the knee, and unloader bracing has been recommended by various guidelines to correct this misalignment. The aim of this report was to provide an update of evidence from the past 10 years on knee braces.

**Methods** MEDLINE/EMBASE search was performed from the past 10 years.

**Results** We reviewed the evidence from 14 published articles. Almost all articles supported knee brace use and showed it to decrease pain, improve function, and improve the quality of life of patients. One study in 2017 followed patients for long term and found knee bracing to be more cost effective than total knee replacement, and could replace the need for surgery. Several minor complications were reported with bracing, like soft tissue irritation, which could be due to poor fitting. A management strategy for this could be regular follow-up at a nurse-led clinic.

**Conclusions** Unloader braces are an economical and effective treatment for unicompartmental knee osteoarthritis. They can significantly improve a patient's quality of life and potentially delay the need for surgery. Patients should be managed with a multidisciplinary approach with conservative management and knee bracing, before surgery is considered.

Osteoarthritis is a common condition with 18% of people over 45 years old seeking treatment for osteoarthritis of the knee joint, 1 and 12% have symptoms associated with osteoarthritis aged 25 to 75 years. 2 Varus or valgus malalignment is a cause of the pathophysiology, specifically for unicompartmental knee osteoarthritis. A varus alignment increases the mechanical load and risk of medial compartmental osteoarthritis, whereas a valgus alignment affects the lateral compartment. 2

The National Institute for Clinical Excellence (NICE) state, in their 2014 guidelines, that treatment for osteoarthritis should take a holistic approach. 3 This should include, at its simplest, patient education and nonpharmacological treatment like exercise, weight loss, aids, and devices such as knee braces, insoles, and walking sticks. 3 Surgical treatments are cost-effective treatments for osteoarthritis and include total knee replacement, unicompartmental knee arthroplasty, and high tibial osteotomy. 4 5 However, waiting lists can be up to 8 months, 6 and there is evidence to suggest that these treatments are not suitable for younger patients suffering from knee osteoarthritis. 7 This is because younger patients tend to have more active lifestyles which increases the risk of any surgical arthroplasty loosening or wearing out. 7 This combined with their longer life expectancy means that they are much more likely to require a revision, 7 especially considering revision rates after 5 years are 6% and 12% after 10 years. 8 Younger patient age groups are more likely to respond better and have improved knee function after total knee replacement; however, a major concern is that they are much more likely to be left dissatisfied with the outcome, especially considering one-fifth of all patients have been reported to be unhappy with their knee after having surgery. 9

NICE guidelines recommend the use of knee braces for the treatment of osteoarthritis, as part of the nonpharmacological management. 3 Osteoarthritis Research Society International have produced guidelines which include biomechanical interventions to treat patients with mild to moderate varus/valgus instability to improve stability and reduce pain. 10 Knee bracing is also incorporated in the European League Against Rheumatism recommendations in 2003, as part of the nonpharmacological management of osteoarthritis. 11

## Knee Bracing

There are different types of knee braces, which can be used to treat knee problems. Depending on the pathology and diagnosis, different types of mechanical support are required. For unicompartmental knee osteoarthritis, unloading type knee braces are more appropriate to unload the affected compartment and realign the knee joint. Studies into knee braces date back into the 20th century. Although the studies of Lindenfeld et al, 12 in 1997, and Kirkley et al, 13 in 1999, are small, they found that valgus knee braces were able to reduce pain and improve function. Also, Katsuragawa et al 14 demonstrated that valgus bracing in patients with medial compartmental osteoarthritis can alter mechanical alignment and increase bone mineral density in the lateral side of the femur and tibia. 14 This suggests there has been an unloading effect from the medial compartment because of the knee brace, and the bone strengthened on the lateral side as it received more load. 14

Out of all the unloading type knee braces, the Unloader One® brace (Ossur) has the most published evidence. It has a 3-point leverage system which is able to unload the affected compartment and is recommended for mild to severe unicompartmental osteoarthritis. 15 The studies by Lindenfeld et al, 12 Kirkley et al 13 and Katsuragawa et al 14 showed that there is some evidence to support unloader knee braces published over 10 years ago. However, in the past 10 years people's lifestyles have changed and the prevalence of osteoarthritis has increased. This along with the fact that surgical techniques have improved and, as a result, expectations have changed, mean an up-to-date review on the past 10 years is required.

The aim of this report was to review previous evidence for unloader knee braces and provide an update on new evidence that has been published in the past 10 years to collate our knowledge on this potential treatment for unicompartmental knee osteoarthritis.

## Methods

A MEDLINE and EMBASE search was performed using search terms including knee, bracing, osteoarthritis, unicompartment*, unloader, valgus, and varus. The search was limited to the English language and the past 10 years. Papers had to be original research published in peer-reviewed journals. Their focus had to be on the unloader knee brace and specifically for their treatment in unicompartmental knee osteoarthritis. Articles had to compare unloader braces to a control or other treatment, or look at a cohort that was using unloader knee braces. One of the main outcomes had to be pain, function, quality of life, knee adduction moments, biomechanics, and gait analysis. The papers were narrowed down based on their titles, abstracts, and then after reading the whole paper by D.M. and P.L. The references and related articles of the papers found were screened for suitable articles. We did not include papers which looked at misalignment after other orthopedic/surgical procedures such as anterior cruciate ligament (ACL) reconstruction.

## Results

The search produced 112 papers, which were narrowed down to 22 based on their titles. Overall, 14 articles were looked at, including 2 papers from 2017. Each paper is presented below and the summary of the evidence is presented in Table 1 .

**Table 1:** Summary of evidence

Study	Type of study	Model	Method	Results	Conclusion
Brouwer et al (2006) 16	In vitro	Multicenter randomized controlled trial	Patients with unicompartmental osteoarthritis were split into two groups—conservative treatment alone or conservative plus brace treatment. They were then assessed for pain severity, knee function score, walking distance, and quality of life. Multilinear regression models were used	117 patients were used and followed up at 12 mo. Brace treatment group outperformed the conservative group in all outcomes. Those who responded better were varus patients, severe osteoarthritis patients, secondary osteoarthritis patients, and < 60 y	Some beneficial effects seen due to knee brace treatment. Some patients did not comply with the brace in the long term
Gaasbeek et al (2007) 17	In vitro	Prospective cohort study	Patients with medial knee osteoarthritis were identified and were given a valgus knee brace for 6 wk. At which point gait analysis and pain and function were assessed	There were improvements in pain and function after 6 wk and gait analysis showed a reduction in varus moment about the knee and this effect was increased in patients with greater deformity	This study identified certain effects knee braces have in a patient's gait. These may have clinical relevance in the future
Ramsey et al (2007) 18	In vitro	Prospective cohort study	Patients were recruited based on a criteria, which included them having medial knee osteoarthritis. Gait analysis was performed without a brace, with a brace in neutral alignment and with the brace in 4° valgus. Pain, instability, and function were assessed using questionnaires	16 patients were recruited. Scores for pain, function, and instability were worst without a brace, but best with the brace in neutral alignment. Cocontractions of paired muscles were reduced with the knee braced, more so with the brace in neutral alignment	Neutral alignment knee braces perform just as well as valgus aligned knee braces in the treatment of medial knee osteoarthritis. This is in regard to reducing pain, disability and cocontraction
Fantini Pagani et al (2010) 19	In vitro	Prospective cohort study	Healthy patients with varus alignment were recruited. Gait analysis of patients were measured with and without a valgus knee brace, in neutral, 4° valgus, and 8° valgus	During walking, there was a significant reduction in knee adduction moments and adduction angular impulse with the brace in 4° valgus and 8° valgus. However, more significant differences were only found when the brace was in 8° valgus when running	Knee braces were effective at reducing knee adduction moments during walking and running, which should aid slowing the progression of osteoarthritis
Schmalz et al (2010) 20	In vitro	Prospective cohort study	Patients were recruited if they had medial knee osteoarthritis. After the subjects had worn the brace for 4 wk, gait analysis and valgus moments of the knee were assessed	There was a reduction in the valgus moment to 10% of it without the brace, which may explain the pain relief experienced by patients. There was a reduction in gait asymmetry when patients were using the brace	Bracing reduced valgus moments around the knee which unloads the medial compartment. This leads to reduction in pain and increase in function
van Raaij et al (2010) 21	In vitro	Randomized controlled trial	Patients with mild medial symptomatic knee osteoarthritis were recruited and randomized into a lateral wedge foot insole group or a valgus brace group. They were assessed at 6 mo on pain severity and function	There were no differences in the improvements in pain and severity after 6 mo in either treatment group. The insole group had better compliance	Lateral wedge foot insoles may be an alternative to valgus bracing to treat medial knee osteoarthritis
Briggs et al (2012) 22	In vitro	Prospective cohort study	Patients were recruited who had been diagnosed with unicompartmental knee osteoarthritis. Patients were instructed to complete the SF-12, WOMAC, and Tegner activity questionnaires, at enrolment, 3 wk, 6 wk, and 6 mo. They also completed an expectation questionnaire at enrolment	39 patients were found, all were prescribed an unloader brace. There was a significant improvement in quality of life, pain, stiffness, and function. Important expectations of these patients included return to recreational sports, improving ability to walk, and pain relief	Unloader braces can be effective treatments to increase function of the knee by reducing pain. This leads to an increase in overall health. Patients' expectations can be met using the brace
Wilson et al (2011) 23	In vitro	Retrospective study	30 patients with unicompartmental knee osteoarthritis were treated with knee braces and followed up at an average of 2.7 y and then again at 11.2 y	24 out of the 30 patients were available to contact. Five patients died but they spoke to the family relatives of these patients. At 2.7 y, 41% were still using the brace, 35% stopped using it, and 24% had undergone arthroplasty. At the second follow-up, 59% had undergone arthroplasty and none of the patients were using the brace	They found the unloader to be effective in the short term but for the long term, most patients preferred to undergo arthroplasty surgery
Larsen et al (2013) 24	Clinical	Prospective cohort study	Patients were recruited with varying degrees of knee osteoarthritis and were tested on walking and sit-to-stand activities after 2 mo of treatment with a knee brace. Pain was also assessed	There were no differences in the improvement of pain between the different grades of osteoarthritis. Sit-to-stand activities were performed better at 1 mo in moderate osteoarthritis and at 2 mo by milder osteoarthritis grades	This study provides data which may help refine the optimum use of knee braces
Niazi et al (2013) 25	Clinical	Prospective cohort study	Patients with symptomatic medial osteoarthritis were identified and were treated with the unloader brace for 6 mo before being assessed for pain, knee function, walking distance, and alignment on X-ray. They were also assessed at baseline	80 patients were enrolled. After 6 mo, there were statistically significant changes in pain and walking distance by using the unloader brace compared with baseline scores	Unloader braces were effective at managing pain relief and improving function in knee osteoarthritis
Iqbal (2014) 26	Clinical	Randomized controlled trial	Patients with symptomatic medial knee osteoarthritis were enrolled. Half were given a knee brace; the other half were given a lateral wedge foot insole. Patients after 6 mo were assessed on pain and function	Pain and walking distance reduced in both treatment groups; however, the unloader brace group outperformed the foot orthotic group. There were slightly more complications with the knee brace	The unloader knee brace is more effective at treating medial knee osteoarthritis than lateral wedge foot insoles
Dessery et al (2014) 27	In vitro	Prospective cohort trial	Patients with medial knee osteoarthritis were asked to wear three different braces—a valgus brace, a valgus and external rotation unloader brace, and an ACL brace. Pain relief, comfort, and gait analysis were performed with each brace	All braces alleviated knee pain but the unloader and ACL brace allowed for a reduction in peak adduction moment which was significant. There was a decrease in gait velocity with the valgus brace. Patients were less inclined to wear the braces due their bulkiness, although the unloader was the most comfortable and had the best compliance rate	When assessing for pain reduction, discomfort, and knee adduction moment, there was little difference between the knee braces
Moyer et al (2017) 28	In vitro	Prospective cohort trial	Patients with medial osteoarthritis were given a valgus brace and lateral wedge foot orthotic. Gait analysis was performed on these patients with no brace or orthotic; with just the brace; with just the orthotic; and then with the orthotic and brace together. During this analysis patients were assessed to walk up and down stairs	There was a reduction in knee adduction moments in all intervention groups during stair descent, more so in the brace and insole group, but no difference in stair ascent. There were less gait decompensations with the combined intervention group too	This suggests that using knee braces and foot orthotics together to correct gait in knee osteoarthritic patients is more effective than using just one but no conclusions can be made as to whether these interventions shift biomechanical load
Lee et al (2017) 1	Clinical	Prospective cohort trial	8 y of data was collected from patients with unicompartmental osteoarthritis. A quality of life questionnaire was collected at enrolment and while wearing the brace. Cost and quality-adjusted life years (QALYs) were then compared with total knee replacement with 8 y of follow-up	There was an average increase of 0.42 after wearing the brace for 26.1 mo in the quality of life questionnaire. Compared with no treatment, the unloader was cost effective at 4 mo or more. At 8 y, the unloader brace had QALY gains of 0.43 and was a lot more cost effective than total knee replacements by £6,467	Unloader knee braces are cost effective for unicompartmental osteoarthritis management. The brace could potentially delay or even replace surgery

Abbreviations: SF-12, 12-Item Short Form Health Survey; WOMAC, Western Ontario and McMaster Universities Osteoarthritis Index.

## Previous Clinical Studies

In 2006, Brouwer et al 16 conducted a randomized controlled trial (RCT) involving 117 patients and reviewed them at 3, 6, and 12 months. Half of the patients were given just conservative management, and the other half knee bracing and conservative management; and they found improvements in knee function and pain in the group using the brace. 16 This study is limited by the fact that patients were not followed up for long term and compliance issues (some patients did not continue to use the brace for more than 3 months). 16 It did see better results in the patients who had varus alignment and in patients under 60 years of age, and therefore they state unloader braces could potentially be used to avoid or delay surgery in the younger patient. 16

Gaasbeek et al 17 in 2007 looked at 15 patients with medial osteoarthritis. The braces were worn for 7 days a week for 6 weeks. 17 They found a decrease in symptoms, as judged by the Western Ontario and McMaster Universities Osteoarthritis Index (WOMAC) scoring system, and a decrease in pain during walking. 17 They also generally found a reduction in peak varus moment around the knee during gait analysis and this effect was more pronounced in patients with worse alignment. 17

Ramsey et al 18 analyzed 16 patients' gaits, with medial knee osteoarthritis, while wearing knee braces. They also looked at the electromyography of muscles around the knee to evaluate the muscle co-contractions (vastus lateralis with lateral hamstrings and vastus medialis with medial hamstrings). 18 They tested patients when unbraced as a baseline, then with bracing to neutral alignment (after wearing for 2 weeks) and with bracing to 4° of valgus (again after wearing for 2 weeks but with a 2-week unbraced period before). 18 Pain and function were also assessed using questionnaires. Nine of the 16 patients reported instability and pain before the study, but this dropped to one after wearing the brace for 2 weeks in neutral alignment. 18 After the washout period and then wearing the brace for 2 weeks in valgus alignment, 6 reported instability. 18 Knee adduction moments during the gait analysis was reduced in both types of bracing and muscle co-contractions significantly improved in both types, but more so when valgus bracing was adopted. 18 Conclusions that can be drawn from this include that unloader braces are effective at alleviating pain, but also that using these braces to align the knee to neutral rather than overcompensating into valgus alignment, may be just as effective. 18

A study performed by Fantini Pagani et al 19 in 2010, was fairly similar to Ramsey et al's 18 study. Fantini Pagani et al 19 found 16 male patients with varus knee alignment and analyzed their gait during walking and running with a valgus brace. The brace was adjusted to neutral, 4° valgus and 8° valgus. 19 They found that the knee brace took away some of the load (due to a reduction in the adduction moments of the knee) during the stance phase of walking and running. 19

Again, Schmalz et al 20 looked at gait analysis in patients with medial osteoarthritis. Schmalz et al 20 had a cohort of 16 patients, all of whom had worn the brace for 4 weeks. During walking, their findings agreed with those of Ramsey et al 18 and Fantini Pagani et al 19 that there is a reduction in knee adduction moments caused by wearing the brace and this, they suggested, could be the mechanism which led to a reduction in symptoms in these patient. They also found that the walking speed increased significantly in their cohort, which again could be due to pain relief.

An RCT by van Raaij et al 21 conducted in 2010 randomized 91 patients with medial knee osteoarthritis, into either a valgus knee brace treatment group or a 10-mm lateral-wedge shoe insole group. Their outcomes included pain, severity, and function. 21 Patients were asked to use the treatment for 6 hours a day every day. 21 After 6 months, there were improvements in pain, severity, and function of the knee in both treatment groups and both performed equally, compared with the baseline data. 21 This paper shows knee braces to be effective in treating some of the symptoms of knee osteoarthritis and improving function. 21 It states that insole wedges could be used as another effective treatment for this condition. 21 Unfortunately, they did not use a control group (no treatment or conservative treatment) which would have allowed assessment of how much each treatment improved pain and function. 21

In 2012, a study was performed looking into the change in quality of life and knee function, if any, before and after 39 patients were treated with a knee brace. 22 Briggs et al 22 followed up their cohort at 3 weeks, 6 weeks, and 6 months. 22 They found an improvement in quality of life, pain, stiffness, and function when patients were given a knee brace. Patients found that they were able to do more recreational activity while being treated with an unloader brace. 22 Briggs et al 22 also looked at the expectations of patients suffering from knee osteoarthritis, as well as investigating unloader knee braces. They identified that two outcomes patients expected as a result of treatment—return to recreational sports and pain reduction (over half of these patients expected all the pain to cease). 22 Other important things were reduction in knee stiffness, knee swelling, and improvement in walking. 22 They summarized patients' expectations as having confidence in the knee, avoiding further deterioration in the future, and maintaining general health. 22

While most of these studies were fairly short term, one of the first long-term studies on knee braces was conducted by Wilson et al 23 in 2011. They only looked at 30 patients, and followed their progress at an average of 2.7 and 11.2 years. 23 The majority of patients reported pain relief, increased function, decreased stiffness, and swelling after exercise and 41% were still using the brace at the first follow-up. 23 However, at 11.2 years, none of the patients were using the knee brace and over half had had a total knee replacement. 23 Of the patients who had a total knee replacement, on average there was 3.9 years between the initial assessment for a knee brace and them having the surgery. 23 They concluded that while the brace, in the short term, was an effective treatment, patient's preferred choice was to have a knee replacement instead of continuing with the brace for long term. 23

In 2013, Larsen et al 24 conducted a prospective study which looked into the effect valgus knee braces have on activities of daily living with their primary focus being walking and sit-to-stand/stand-to-sit activities. Their patients had been wearing the brace for 2 months during the study. 24 In patients with low and moderate knee osteoarthritis, they found an increase in activity while using the brace. 24 This was coupled with a decrease in pain too, which may have contributed to the increase in activity level. 24 With regards to walking, their patients had a nonsignificant improvement in knee adduction but they found that they were able to push off with more power. 24 This suggests that the brace, by redistributing the loading forces in the knee, is aiding the strengthening of muscles in the leg. 24 This then leads to increased function and activity levels found in this study, including improvements in sit-to-stand and stand-to-sit exercises. 24 Overall, they could only conclude that valgus bracing in medial knee osteoarthritis could lead to short-term improvements in function of the knee in patients with mild to moderate osteoarthritis. 24 Unfortunately, they did not look at patients with severe osteoarthritis and their study only lasted 2 months, so no long term conclusions could be drawn from this; however, they did state that the brace has the potential to delay to avoid surgical intervention in these patients. 24

Niazi et al 25 in 2013 conducted a case series study to assess any change in pain and function in the knee in 80 patients with medial knee osteoarthritis using an unloader knee brace. Their outcomes included pain severity, walking distance, and knee function scores (using WOMAC scoring system). 25 Four patients were lost to follow-up. 25 Pain severity and walking distance significantly improved in these patients after 6 months of brace use and they also found that patients used less pain killers as a result and knee function scores improved. 25 They stated that unloader bracing is an effective treatment for unicompartmental knee osteoarthritis and can be used in patients who are less keen on or contraindicated against surgery. 25 Niazi et al 25 concluded that it should be considered in all patients with this condition along with standard medical treatment, and surgical intervention should not be considered unless a patient has tried to use an unloader brace.

Iqbal 26 (part of a similar team to Niazi et al 25 ) conducted an RCT comparing unloader knee braces with lateral wedged insoles, 26 similar to van Raaij et al. 21 In total, 120 patients were involved in the study by Iqbal. 26 Patients were instructed to wear the brace for 3 to 4 hours for the first week then as long as they could during the day after that. 26 Their main outcomes, like their previous paper, was pain and walking distance (using the second section of the Lequesne scale). 26 Four patients using insoles and two using braces were lost to follow-up and three in the insole group and two in the brace group changed treatment because of lack of symptomatic relief or complications. 26 The baseline differences in pain and severity between the two groups were insignificant; however, after 6 months of treatment, the brace group's pain score was 3.97 and the insole group's score was 4.53. 26 For walking distance, the brace group was also better with a score of 1.93 on the Lequesne scale—an improvement of 0.43 compared with the lateral wedge insole group, and there was an improvement in activity levels in the braced group; these differences between the two groups were statistically significant. 26 It was concluded by Iqbal 26 that despite improvements in both groups, the unloader knee brace outperformed lateral wedge insoles in all outcomes; however, there were a few more complications associated with bracing; five patients experienced leg swelling.

Dessery et al 27 looked at 24 patients with medial knee osteoarthritis, each tried three different knee braces for 2 weeks before assessment. They assessed pain relief, comfort, and gait analysis. 27 The three braces in question were a valgus brace, an unloader brace with valgus and external rotation functions, and a functional knee brace for ligament injuries (ACL-brace). 27 All three braces alleviated pain immediately but the unloader brace and ACL brace allowed for a significant decrease in load during the stance phase. 27 The valgus brace saw a decrease in gait velocity. 27 They only show pain relief differences from the braces in the short term and focused more on the biomechanical analysis of the gait. 27 They also reported patients were less inclined to wear the braces for long periods due to their bulkiness but the unloader brace was the most comfortable of the three. 27 In conclusion, they stated that all three braces were similar in terms of pain and function improvements. 27

## Most Recent Research in 2017

Moyer et al 28 looked at 35 patients' gait during stair ascent and descent. At first, the patients were tested with no mechanical aids, then with just a custom-fit valgus knee brace, then a lateral wedge foot insole, and then both the brace and foot insole. 28 During stair descent, there was a reduction in knee adduction moments in all intervention groups compared with the control, more so in the brace and insole group, but no difference in stair ascent. 28 Gait speeds were similar across all conditions during both ascent and descent. 28 Overall, there were fewer gait compensations during descent compared with ascent. 28 In summary, the knee brace and lateral wedge insole had the best biomechanical effect on gait during stair descent and almost half the patients (17 patients) preferred this treatment, although it did not have the same effect on stair ascent. 28 This study again looked at a very little number of patients and did not look at patients over the long term which makes it hard to clinically relate this study to practise. 28

A prospective study conducted by Lee et al 1 followed patients up for over 8 years while they were treated with the Ossur Unloader One knee brace. It is one of the first studies to provide long-term evidence about unloader knee brace use and followed 63 patients with end-stage knee osteoarthritis (irrespective of the affected compartment)—one patient withdrew. 1 Patients on average wore the brace for 26 months, with some opting out of surgery and instead preferring to use the knee brace for long term. 1 At the final follow-up, 40% of patients did not require surgery; excluding these, of the remaining 38, the average brace use was 8.6 months. 1 Their analysis showed that if patients were able to tolerate the unloader brace for 2 years their chances of having surgery decreased significantly; in fact, in the study, anyone who wore the brace for more than 2 years, did not go on to have surgery. 1 Wearing the brace for 6 months halved the chance of a patient having to need surgery compared with those who wore the brace for 3 months or less. 1 Their results suggested that unloader knee braces are cost effective in patients who are awaiting surgery. 1

Analysis of their results showed that there was no difference in how long patients wore the brace, depending on certain patient demographics including gender, age, body mass index (BMI), socioeconomic group, leg, or compartment. 1 So, potentially all patients could benefit from unloader braces if they have unicompartmental osteoarthritis. Overall, Lee et al 1 showed the unloader knee brace to be a cost-effective management option for unicompartmental osteoarthritis, as it can delay or reduce the frequency of surgery.

A summary of this evidence can be found in Table 1 .

## Discussion

The aim of this review was to give an update on the literature in the past 10 years on unloader braces including the more recent articles in 2017. We presented 14 pieces of original research. Lee et al's 1 study was the only piece that looked at a large cohort of patients over the long term to investigate unloader knee brace use. They found that knee braces were cost effective but importantly could replace the need for surgery, if not delay it over 8 years. The other long-term study, by Wilson et al, 23 was retrospective and only used 30 patients, so while their results are important, their conclusions are not as strong as Lee et al's. 1

The majority of studies also agreed that unloader braces were a valuable treatment for unicompartmental knee osteoarthritis, whether that be in reducing pain or looking more specifically at the knee adduction moments during walking. Unfortunately, many have short follow-up periods, small sample sizes, and most focus on medial compartment osteoarthritis. This means we cannot draw similar conclusions from these studies like we can in Lee et al's, 1 such as the fact that unloaders are able to delay surgery over the long term. Only three papers were RCTs, 16 21 26 which carry the highest level of evidence. All three showed patients benefitted from unloader brace treatment compared with conservative management.

Our results concurred with other literature studies, so are not novel findings but reinforce the same conclusions. Ramsey and Russel, 29 in 2009, concluded that unloader knee braces are good at helping pain relief in unicompartmental knee osteoarthritis. With regards to practice, it should be used in conjunction with other conservative treatment and be considered before any type of surgical intervention, a point emphasized by Lee et al 1 and Niazi et al. 25 A meta-analysis conducted by Moyer et al 30 in 2015 looked at the evidence presented in eight RCTs on valgus knee bracing for medial compartmental osteoarthritis. They concluded that they were able to make a strong recommendation about improvements with pain in patients with valgus knee braces and a weak recommendation for improvement in function. 30 Petersen et al 31 focused on knee adduction moments and 20 out of the 24 articles they looked at concluded that unloader braces reduced the knee adduction moment. The evidence from this review and others combined should be enough to encourage further the use of knee unloader braces in clinical practice.

Wilson et al 23 determined that patients preferred to opt for surgery over knee braces in the long term, although this is in disagreement with Lee et al, 1 which found long-term use of the brace could delay or potentially nullify the need for surgery. One of the reasons for lower rates of use, especially long term, could be due to the complications of using knee braces. Lee et al 1 reported that 43% of patients in their study had soft tissue injuries which could be due to poor fitting. Moyer et al, 30 in their meta-analysis, found compliance rates varied from 45 to 100%. Minor complications mentioned in this review include that the brace was bulky; its fitting was constraining or it slipped; and it caused swelling, blistering, and skin irritation. 30 Iqbal 26 reported 5 out of the 60 patients wearing bracing reported leg swelling. With regards to major complications, there have been very few reported, although there have been cases of thrombophlebitis and venous thromboembolism. 32 33

To improve compliance, Lee et al 1 suggested regular follow-up appointments, in a nurse-led clinic, at 1, 3, and 6 months to address any issues. This way, complications can be monitored and the health care service can check the patient is fitting the brace correctly, which was suggested as the cause for many soft tissue issues. If there were any problems with fitting, these could be corrected to avoid worsening of the problem and increasing the chances of successful treatment. 1

There were several studies present which also looked at lateral wedge foot insoles as a potential treatment for unicompartmental knee osteoarthritis too. While two studies suggested that they could be as good as knee braces, 21 28 Iqbal's 26 RCT showed that knee braces outperformed foot insoles in improvements in pain and walking distance, so they may not be as suitable, despite the fact they had slightly less complications. More patients in Iqbal's 26 study wanted to change from foot insoles due to lack of effect, than wanted to change to knee braces.

Briggs et al 22 identified that two outcomes that patients expected as a result of treatment were return to recreational sports and pain reduction. From the evidence above, it shows that knee bracing may be able to manage these expectations (fully or partially), but it is surprising that there have been reports that unloader knee braces are not regularly prescribed in clinical practice. A study by Li et al 34 found that less than 12% of patients, suffering from unicompartmental osteoarthritis, had tried knee bracing as part of their treatment. Although this study was in 2004, it is backed up by two more recent studies showing big inconsistencies between clinical practice and guidelines for knee osteoarthritis management. 32 35

Future research needs to look into the disparity in volume of evidence between medial and lateral unicompartmental knee osteoarthritis. Most of the studies in this review were also short term (around 6 months) and more evidence is required to validate unloader braces over a longer period of time. Two studies did look at the braces over 8 years 1 and 11 years, 23 but if unloader braces can actually delay the need for surgery then they need to be effective over at least a 5-year period and the evidence needs to reflect this. Also, there were very few studies looking at knee brace use for end-stage knee osteoarthritis, so more research may be needed here to see how exactly they could be used for this stage of the condition. More research will be needed to identify the optimal patient who would benefit from unloader knee braces (with regards to gender, age, BMI, compartment, etc.), although there are suggestions that every patient could benefit. A limitation from this study include that we looked at a mix of studies with different outcomes, so they were slightly harder to compare.

## Conclusion

Current research has demonstrated the effectiveness of unloader braces in the management of unicompartmental knee osteoarthritis over the short term; only two papers studied the braces over a long period (8 and 11 years). Unloader braces are a cost-effective management option to potentially delay the need for surgery for young patients or those on long waiting lists. It has been shown to dramatically affect a patient's quality of life and should be combined with other standard treatments as stated in the guidelines for knee osteoarthritis management. More investigations are needed into why prescription rates are low. Although we have explored some complications and issues with compliance, these issues do not justify not considering an unloader brace in every patient presenting with unicompartmental knee osteoarthritis. One reason for this may be lack of awareness, and therefore more education on unloader braces may be required. A nurse-led clinic for follow-up has been demonstrated to be an effective approach to monitor compliance and complications. These conclusions are not novel but support existing literature and this review can be added to the ever-growing pool of evidence on the use of unloader braces. This evidence combined with previous studies and reviews should lead to an increased uptake, acceptance, and usage of the braces.

Patients with unicompartmental knee osteoarthritis should be managed with a multidisciplinary approach, an unloader knee brace, standard conservative management plus a follow-up clinic lead by nurses; with the overall aim being to aid patients' quality of life and to reduce the rates of knee replacements.
